# Clinical recommendations for perioperative immunotherapy‐induced adverse events in patients with non‐small cell lung cancer

**DOI:** 10.1111/1759-7714.13942

**Published:** 2021-03-30

**Authors:** Jun Ni, Miao Huang, Li Zhang, Nan Wu, Chun‐Xue Bai, Liang‐An Chen, Jun Liang, Qian Liu, Jie Wang, Yi‐Long Wu, Feng‐Chun Zhang, Shu‐Yang Zhang, Chun Chen, Jun Chen, Wen‐Tao Fang, Shu‐Geng Gao, Jian Hu, Tao Jiang, Shan‐Qing Li, He‐Cheng Li, Yong‐De Liao, Yang Liu, De‐Ruo Liu, Hong‐Xu Liu, Jian‐Yang Liu, Lun‐Xu Liu, Meng‐Zhao Wang, Chang‐Li Wang, Fan Yang, Yue Yang, Lan‐Jun Zhang, Xiu‐Yi Zhi, Wen‐Zhao Zhong, Yu‐Zhou Guan, Xiao‐Xiao Guo, Chun‐Xia He, Shao‐Lei Li, Yue Li, Nai‐Xin Liang, Fang‐Liang Lu, Chao Lv, Wei Lv, Xiao‐Yan Si, Feng‐Wei Tan, Han‐Ping Wang, Jiang‐Shan Wang, Shi Yan, Hua‐Xia Yang, Hui‐Juan Zhu, Jun‐Ling Zhuang, Ming‐Lei Zhuo

**Affiliations:** ^1^ Peking Union Medical Hospital Chinese Academy of Medical Science & Peking Union Medical College Beijing China; ^2^ Peking University Cancer Hospital Beijing China; ^3^ Zhongshan Hospital Fudan University Shanghai China; ^4^ The First Medical Center of Chinese PLA General Hospital Beijing China; ^5^ Peking University International Hospital Beijing China; ^6^ Chinese Journal of Lung Cancer Beijing China; ^7^ National Cancer Center, National Clinical Research Center for Cancer Cancer Hospital, Chinese Academy of Medical Sciences and Peking Union Medical College Beijing China; ^8^ Guangdong Lung Cancer Institute, Guangdong Provincial People's Hospital, Guangdong Academy of Medical Sciences Guangzhou China; ^9^ Fujian Medical University Union Hospital Fuzhou China; ^10^ Tianjin Medical University General Hospital, Tianjin Key Laboratory of Lung Cancer Metastasis and Tumor Microenvironment Tianjin Lung Cancer Institute Tianjin China; ^11^ Shanghai Chest Hospital Shanghai Jiao Tong University Shanghai China; ^12^ The First Affiliated Hospital Zhejiang University School of Medicine Zhejiang China; ^13^ Tangdu Hospital Fourth Military Medical University Xi'an China; ^14^ Ruijin Hospital Shanghai Jiao Tong University School of Medicine Shanghai China; ^15^ Union Hospital, Tongji Medical College Huazhong University of Science and Technology Wuhan China; ^16^ China‐Japan Friendship Hospital Beijing China; ^17^ Cancer Hospital of China Medical University Liaoning Cancer Hospital & Institute Shenyang China; ^18^ Jilin Cancer Hospital Changchun China; ^19^ West China Hospital, Sichuan University Chengdu China; ^20^ National Clinical Research Center for Cancer, Key Laboratory of Cancer Prevention and Therapy, Tianjin's Clinical Research Center for Cancer Tianjin Medical University Cancer Institute and Hospital Tianjin China; ^21^ Peking University People's Hospital Beijing China; ^22^ Sun Yat‐sen University Cancer Center Guangzhou China; ^23^ Xuanwu Hospital, Capital Medical University Beijing China

**Keywords:** clinical recommendation, irAE, non‐small cell lung cancer, perioperative immunotherapy

## Abstract

Perioperative adjuvant treatment has become an increasingly important aspect of the management of patients with non‐small cell lung cancer (NSCLC). In particular, the success of immune checkpoint inhibitors, such as antibodies against PD‐1 and PD‐L1, in patients with lung cancer has increased our expectations for the success of these therapeutics as neoadjuvant immunotherapy. Neoadjuvant therapy is widely used in patients with resectable stage IIIA NSCLC and can reduce primary tumor and lymph node stage, improve the complete resection rate, and eliminate microsatellite foci; however, complete pathological response is rare. Moreover, because the clinical benefit of neoadjuvant therapy is not obvious and may complicate surgery, it has not yet entered the mainstream of clinical treatment. Small‐scale clinical studies performed in recent years have shown improvements in the major pathological remission rate after neoadjuvant therapy, suggesting that it will soon become an important part of NSCLC treatment. Nevertheless, neoadjuvant immunotherapy may be accompanied by serious adverse reactions that lead to delay or cancellation of surgery, additional illness, and even death, and have therefore attracted much attention. In this article, we draw on several sources of information, including (i) guidelines on adverse reactions related to immune checkpoint inhibitors, (ii) published data from large‐scale clinical studies in thoracic surgery, and (iii) practical experience and published cases, to provide clinical recommendations on adverse events in NSCLC patients induced by perioperative immunotherapy.

## Graphical abstract

Perioperative irAE management is an important part of immune perioperative treatment of lung cancer. With the continuous development of immune perioperative treatment, more research is needed in the future to optimize the diagnosis and treatment of perioperative irAE.

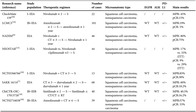

## INTRODUCTION

Immune checkpoint inhibitors (ICIs) promote antitumor immunity by preventing inhibitory signaling through checkpoint receptors such as programmed death‐1 (PD‐1) and cytotoxic T lymphocyte‐associated antigen‐4 (CTLA‐4) expressed on T lymphocytes and their ligands programmed death ligand‐1 (PD‐L1) and CD80/CD86, respectively, expressed on tumor cells and other immune cells.^1^, ^2^ ICIs have revolutionized the treatment of many cancers, including non‐small cell lung cancer (NSCLC). Monoclonal antibodies that target the PD‐1–PD‐L1 axis (e.g., nivolumab, pembrolizumab, atezolizumab, and durvalumab) have shown efficacy in advanced NSCLC^3^ and have been approved as first‐ and second‐line treatment in many countries. In the 2017 PACIFIC study of patients with stage III unresectable NSCLC, patients treated with durvalumab after concurrent chemotherapy and radiotherapy survived significantly longer than those treated with radiotherapy and chemotherapy alone. The PACIFIC treatment regimen has become standard for stage III unresectable NSCLC.^4^ Many additional clinical studies of perioperative immunotherapy are now being carried out to improve the cure rate and prolong disease‐free survival (DFS) of patients with locally advanced NSCLC.^5^ The goal of preoperative immunotherapy, which includes ICI monotherapy, double therapy, and chemotherapy combined with immunotherapy, is to prevent suppression and/or induce reactivation of antitumor T cells, thereby reducing the disease stage, improving the rate of complete (R0) resection, controlling microsatellite foci, and improving the overall survival (OS) rate. Phase II/III clinical studies of postoperative adjuvant immunotherapy are also underway. Among them, the NADIM and SAKK 16/14 studies^6^, ^7^ of 46 and 68 patients, respectively, with early/locally advanced NSCLC found that perioperative chemotherapy plus immunotherapy resulted in a major pathological response (MPR) rate of 60%–85%, and a complete pathological response (pCR) rate of 18.2%–71.4%. In addition, a similar study of neoadjuvant immunotherapy for stage IIIA/B NSCLC showed a significant correlation between OS and both pCR and MPR,^8^ suggesting that perioperative immunotherapy may be an excellent choice for NSCLC treatment in the future.

Despite the clinical benefits of immunotherapy, it is inevitable that adverse events (AEs) will occur. Such immunotherapy‐related AEs (irAEs) can be serious, and in a few cases even life‐threatening, resulting in inoperability, delayed surgery, and increased postoperative complications.^9^ A meta‐analysis^9^, ^10^ conducted by Lung Adjuvant Cisplatin Evaluation (LACE) and NSCLC Collaborative Group showed that neoadjuvant immunotherapy reduced the risk of death in NSCLC patients by 13%. Grade 3–4 AEs were as high as 66% for both therapies; however, irAEs of pneumonia, cardiac toxicity, digestive tract toxicity, and other rare but serious toxicities seriously affected patient prognosis.^11^ There is thus an urgent need for better management of irAEs, and specific steps should be embodied in procedures for baseline examination, diagnosis, differential diagnosis, and multiple disciplinary management models.

Lung cancer treatment has entered a new era, and thoracic surgery plays a vital role in the clinical application immunotherapy and the management of its adverse reactions. At present, doctors in the field of lung cancer diagnosis and treatment, especially thoracic surgeons, are facing many challenges such as reorganization of knowledge structure, and increased professional extensibility. To address these challenges, in this review, we have integrated the opinions of clinicians in many disciplines and put forward general clinical recommendations that aim to provide a relevant knowledge structure, provoke opinions, improve the conceptual diagnosis and treatment of perioperative irAEs, and provide more effective clinical guidance.

While the specific mechanisms underlying many irAEs is unclear, several potential mechanisms have been proposed, including (i) enhanced activity of T cells against antigens expressed by tumors and normal tissues, (ii) activity of existing and new autoimmune antibodies, (iii) increased production of inflammatory cytokines, and (iv) in the case of CTLA‐4–CD80/CD86 checkpoint blockade, immune responses promoted by direct binding of anti‐CTLA‐4 antibody to normal cells expressing CTLA‐4.^12^Immune attack of normal tissues can cause a range of irAEs, the most common being toxicities of the skin, endocrine system, liver, gastrointestinal tract, lungs, and skeletal muscle, and transfusion reactions. Less commonly, irAEs may also affect the nervous system, blood, kidneys, heart, and eye.^12^

Based on the unique mechanism of action of ICIs, the incidence, severity, and type of irAEs differ from AEs associated with traditional chemotherapy. One report^13^ noted that patients receiving immunotherapy had significantly lower rates of any grade AEs (65.8% vs. 85.2%, odds ratio [OR] 0.35), grade ≥3 AEs (16.5% vs. 41.1%, OR 0.26), rate of treatment interruption due to AEs (6.4% vs. 10.8%, OR 0.55, 95% confidence interval 0.39–0.78), and rate of death due to treatment‐related AEs (0.87% vs. 1.28%) compared with patients receiving chemotherapy. The most common irAEs of any grade were (in order of frequency) diarrhea followed by hypothyroidism, elevated aspartate aminotransferase (AST), vitiligo, and elevated alanine aminotransferase (ALT), while the most common irAEs of grade ≥3 were elevated AST and ALT, pneumonia, diarrhea, and colitis.^15^ irAEs usually emerge weeks to months after initiation of ICI therapy and are generally of long duration, sometimes lasting until and beyond the end of treatment.

Several phase III clinical studies are currently investigating perioperative immunotherapy for NSCLC patients, including the CheckMate‐816 (NCT02998528), KEYNOTE‐671 (NCT03425643), IMpower‐030 (NCT03456063), and AEGEAN (NCT03800134) trials. These studies have reported MPRs of 19%–45% for neoadjuvant ICI monotherapy, ~33% for combination ICI therapy, and as high as 85% for ICI combined with chemotherapy. Phase I/II clinical studies have also demonstrated the beneficial potential of perioperative immunotherapy, although several studies^6^, ^14^, ^15^, ^16^ reported AEs resulting in delayed surgery, altered surgery mode, reduced operative benefit, prolonged hospital stay, and increased economic burden on patients. Moreover, the incidence of perioperative complications and mortality was increased for patients with severe AEs. Therefore, careful management of lung cancer patients receiving perioperative immunotherapy should include early identification of irAEs and intervention with immunosuppressive and/or immunomodulatory agents. Guidelines from authorities such as the Chinese Society of Clinical Oncology and National Comprehensive Cancer Network (NCCN) have pointed out that prevention, assessment, examination, treatment, and monitoring of the reactions are all essential components in the overall process of irAE management.

## PREVENTION OF IRAES

### Immunotherapy for special patient populations

Although the utility of immunotherapy for operable NSCLC is currently being explored, support from higher‐level evidence‐based medicine is needed. The results of completed studies are exciting, but the number of cases included is limited, and the high disease control rate needs to be further confirmed by larger‐scale clinical studies. The possible benefits and potential risks of immunotherapy must be fully explored in clinical practice, especially in special patient populations such as those with autoimmune diseases, organ dysfunction, and transplant‐associated immunosuppression to ensure the best outcomes of perioperative immunotherapy and of the surgery itself.

#### Patients with autoimmune diseases

ICI treatment can lead to recurrence or exacerbation of existing autoimmune diseases^17^, ^18^and may even elicit new ones.^19^ Therefore, immunosuppressed patients who are administered ICIs should be closely monitored by specialists. Before starting immunotherapy, we suggest that the dose of prednisone is reduced to <10 mg/day (or equivalent). Patients who are not suitable for immunotherapy include those with autoimmune nervous system diseases or any life‐threatening autoimmune disease, particularly if their disease cannot be controlled by immunosuppressive drugs or they require large doses of immunosuppressive drugs to maintain control.

#### Organ and hematopoietic stem cell transplantation recipients

ICI therapy may lead to graft‐versus‐host disease (GVHD) or failure of organ transplants. The incidence of GVHD in kidney, liver, and heart transplant patients is about 50%, 44%, and 25%, respectively. Before initiating immunotherapy, the possible outcomes should be fully discussed with the patients and transplant surgeons. Patients who have previously received solid organ transplants and have a feasible alternative treatment if and when graft rejection occurs may be suitable for immunotherapy if there is no evidence of transplant rejection and they are in the maintenance treatment stage of immunosuppression.

#### Patients with chronic viral infection

Because the interaction between ICIs and chronic viral infection is unclear, patients chronically infected with hepatitis B virus (HBV), hepatitis C virus (HCV), or human immunodeficiency virus (HIV) have been excluded from clinical trials to date. However, ICI therapy is currently considered to be safe and effective in uninfected patients with hepatocellular cancer.^20^, ^21^A few studies have reported that ICI treatment reduces the CD4+ T lymphocyte count in HIV‐infected patients, rendering them immunodeficient. Moreover, ICIs have little effect and can induce immune reconstitution inflammatory syndrome.^22^

#### Selection of patients for perioperative immunotherapy

As noted earlier, ICIs such as monoclonal antibodies against PD‐1, PD‐L1, and CTLA‐4 have proven useful in the treatment of locally advanced or metastatic NSCLC, and clinical trials of adjuvant/neoadjuvant immunotherapy for operable NSCLC are underway (Tables 1 and 2).

**TABLE 1:** Summary of phase I/II clinical trials of neoadjuvant immunization combined with or without chemotherapy for resectable non‐small cell lung cancer (NSCLC)

Research name (reference)	Study population	Therapeutic regimen	Number of cases	Microanatomy type	EGFR	ALK	PD‐L1	Main results
CheckMate 159^[26]^	I–IIIA	Nivolumab × 2 → S	22	Squamous cell carcinoma, nonsquamous carcinoma	/	/	/	MPR: 45% pCR:15%
LCMC3^[14]^	IB–IIIA	Atezolizumab × 2 → S → atezolizumab × 1 year	101	Squamous cell carcinoma, nonsquamous carcinoma	WT	WT	+/−	MPR:19% pCR: 5%
NADIM^[6]^	IIIA	Nivolumab + CT × 3 → S → Nivolumab × 1 year	46	Squamous cell carcinoma, nonsquamous carcinoma	WT	WT	+/−	MPR: 80% pCR:75%
NEOSTAR^[25]^	I–IIIA	Nivolumab vs. Nivolumab+Ipilimumab ×3 → S	44	Squamous cell carcinoma, nonsquamous carcinoma	/	/	/	MPR: 17% vs. 33% (ITT) pCR: 9% vs. 29% (ITT)
NCT03366766^[27]^	I–IIIA	Nivolumab + CT × 3 → S	13	Squamous cell carcinoma, nonsquamous carcinoma	WT	WT	+/−	MPR:85% pCR:38%
SAKK 16/14^[7]^	IIIA	CT × 3 → durvalumab × 2 → S→ durvalumab × 1 year	68	Squamous cell carcinoma, nonsquamous carcinoma	WT	WT	+/−	MPR: 60% pCR:18.2%
ChiCTR‐OIC‐17013726^[16]^	IB–IIIB	Sintilimab × 2 → S → Sintilimab ± CT/CT ± RT	40	Squamous cell carcinoma, nonsquamous carcinoma	WT	WT	+/−	MPR: 40.5% pCR:16.2%
NCT02716038^[28]^	IB–IIIA	Atezolizumab + CT × 4 → S	39	Squamous cell carcinoma, nonsquamous carcinoma	/	/	+/−	MPR:57% pCR: 33%

Abbreviations: CT, chemotherapy; MPR, major pathological response; pCR, pathological complete response; S, surgery; WT, wild‐type.

**TABLE 2:** Summary of phase III clinical trials of neoadjuvant immunotherapy combined with chemotherapy in resectable non‐small cell lung cancer (NSCLC)

Research name	Study population	Therapeutic regimen	Number of cases	Microanatomy type	EGFR	ALK	PD‐L1	Estimated completion time
IMpower030 (NCT03456063)	II–IIIB	CT+ atezolizumab/placebo × 4 → S→ atezolizumab/placebo × 1 year	374	Squamous cell carcinoma, nonsquamous carcinoma	WT	WT	+/−	March, 2025
AEGEAN (NCT03800134)	IIA–IIIB	CT + durvalumab/placebo×3 → S → durvalumab/placebo ×1 year	300	Squamous cell carcinoma, nonsquamous carcinoma	WT/m	WT/m	+/−	January, 2024
KEYNOTE‐671 (NCT03425643)	II–IIIB	CT + pembrolizumab/placebo ×4 → S → pembrolizumab/placebo × 1 year	786	Squamous cell carcinoma, nonsquamous carcinoma	/	/	+/−	June, 2026
CheckMate 77 T (NCT04025879)	IIA–IIIB	CT + Nivolumab/placebo→S→ Nivolumab/placebo × 1 year	452	Squamous cell carcinoma, nonsquamous carcinoma	WT	WT	+/−	September, 2024
CheckMate 816 (NCT02998528)	IB–IIIA	CT + nivolumab × 3 → S vs. CT × 3 → S	350	Squamous cell carcinoma, nonsquamous carcinoma	/	/	+/−	May, 2023

Abbreviations: CT, chemotherapy; m, mutation; S, surgery; WT, wild‐type.

Studies of neoadjuvant immunotherapy have mainly focused on patients with early stage (IB–IIIB) NSCLC, and no guidelines for their use as auxiliary therapy have yet been published. Patients enrolled in the clinical study can follow the screening process of the study. At present, NSCLC patients selected for neoadjuvant immunotherapy in real‐world clinical practice tend to have high T stage, multiple N2 metastasis, and fusion N2 metastasis, and earlier disease stages are still being explored.The current NCCN guidelines recommend postoperative adjuvant therapy (chemotherapy, radiotherapy, or targeted therapy) for patients with completely resected (R0) NSCLC but not for patients with stage IA NSCLC; it may be considered for patients with stage IB NSCLC with high risk factors, although the recommendations lack high‐level evidentiary support. Ongoing clinical trials of postoperative adjuvant immunotherapy based on the recommendation of the guidelines mainly include patients with completely resected stage IB–IIIA NSCLC (Table 3).

**TABLE 3:** Summary of phase III clinical trials of adjuvant immunotherapy after resectable non‐small cell lung cancer (NSCLC)

Research name	Study population	Therapeutic regimen	Number of cases	Microanatomy type	EGFR	ALK	PD‐L1	Estimated completion time
IMpower010 (NCT02486718)	IB–IIIA	CT × 4 → atezolizumab/placebo × 1 year	1280	Squamous cell carcinoma, nonsquamous carcinoma	/	/	+/−	December 2027
ALCHEMIST‐nivo/ANVIL (NCT02595944)	IB–IIIA	Nivolumab/observation×1 year ± CT/RT	903	Squamous cell carcinoma, nonsquamous carcinoma	WT	WT	+/−	July 2024
PEARLS/KEYNOTE‐091 (NCT02504372)	IB/II–IIIA	Pembrolizumab/placebo × 1 year ± CT	1177	Squamous cell carcinoma, nonsquamous carcinoma	/	/	+/−	February, 2024
ADJUVANT BR.31 (NCT02273375)	IB–IIIA	Durvalumab/placebo × 1 year	1360	Squamous cell carcinoma, nonsquamous carcinoma	/	/	+/−	January, 2024
ALCHEMIST Chemo‐IO (NCT04267848)	IB–IIIA	CT × 4 vs. CT × 4 + pembrolizumab × 1 year vs. CT × 4 → pembrolizumab × 1 year	1263	Squamous cell carcinoma, nonsquamous carcinoma	WT	WT	+/−	December 2024

Abbreviations: CT, chemotherapy; RT, radiotherapy; WT, wild‐type.

For patients with incomplete resection (R1 or R2) after stage IB NSCLC, maintenance immunotherapy can be considered after postoperative adjuvant therapy if a second operation is not considered. However, there are no current guidelines for the use of ICIs as auxiliary therapy and they are not routinely used in this context at present.

### Assessment of irAEs

Evaluation and routine screening of NSCLC patients before initiation of immunotherapy may be the most important component of irAEs management because it allows the patients likely to be most susceptible to irAEs to be identified and flagged for early intervention. Before starting ICI treatment, physicians should assess current medical history, past medical history (especially autoimmune disease, immunodeficiency disease, and special infection history), personal and family history, and general condition, and perform baseline laboratory and imaging examinations (e.g., basic chest and abdomen computed tomography, magnetic resonance imaging of the head) (Table 4). All patients should be informed of the potential for immunotherapy to induce adverse reactions. When AEs occur, patients should be advised to report symptoms promptly and directly to the treatment team. Timely treatment of irAEs is needed to prevent exacerbation or deterioration of the patient's condition.

**TABLE 4:** Baseline assessment

Inspection item	Class I recommendation	Class II recommendation	Class III recommendation
Clinical evaluation^a^	Physical examination Autoimmune diseases or organ‐specific diseases, endocrine diseases or infectious diseases Nervous system assessment Bowel evacuation habit Smoking history, family history, pregnancy status Baseline drug use		
Imaging evaluation^b^	Chest and abdomen (pelvic cavity) enhanced CT	Whole body PET/CT	Head MRI and whole body bone scan were performed according to clinical indications
General hematology test^c^	Routine blood test Blood biochemistry Blood coagulation Myocardial enzyme Urine routine Then routine Inflammation index	Patients with elevated blood sugar need to improve the urinary ketone body, glycosylated hemoglobin, insulin and C peptide	Improve insulin autoantibodies (IAA), islet cell antibodies (ICA) and glutamic acid decarboxylase antibodies (GAD‐Ab) according to the general condition
Virology test	Five hepatitis B HIV‐Ab, TP‐Ab CMV‐DNA EBV‐DNA Novel coronavirus antibody (IgM + IgG)		
Autoantibodies^d^	ANA spectrum ANCA spectrum Rheumatoid associated antibody Antibody against acetylcholinesterase Anti‐Hu/Yo/Ri antibody (blood + cerebrospinal fluid) (for patients with small cell lung cancer)		
Skin	In case of new skin lesions or aggravation of existing skin lesions, skin and mucosa (conjunctiva, oral mucosa, nasal mucosa, perianal mucosa, etc.) should be examined		
Pancreas	Blood amylase and lipase	If there are symptoms, consider abdominal enhanced CT (pancreatic thin scan) or MRCP	
Thyroid gland^e^	Thyroid function test: Thyroid stimulating hormone (TSH), free thyroxine (fT4), free triiodothyronine (fT3)	If the baseline thyroid function is abnormal, check TT3 and TT4, fT3 and fT4, antiperoxidase antibody (TPO), antithyroglobulin antibody (TgAb) and thyrotropin receptor antibody (TRAb)	
Adrenal gland/pituitary gland^e^	Blood cortisol (preferred in the morning, 8:00 a.m.), adrenocorticotropic hormone (ACTH) (8:00 a.m.)	If the baseline examination is abnormal, improve six sex hormones (LH [luteinizing hormone], FSH [follicle stimulating hormone], T [testosterone], P [progesterone], E2 [estradiol], PRL [prolactin]) and insulin‐like growth factor (IGF‐1); Pituitary MRI	
Lung	Oxygen saturation (resting and active) Routine pulmonary function test (PETs) was performed before operation, and blood gas analysis was performed in high‐risk patients	6 min walking test (6MWT) is recommended	
Heart and blood vessels	Electrocardiographic examination Heart color Doppler ultrasound	For patients with abnormal baseline or symptoms, regular monitoring, individualized follow‐up with cardiology consultation as needed	
Skeletal muscles		Joint examination/functional evaluation as required	C‐reactive protein (CRP), ESR, creatine phosphokinase (CPK), muscle zymogram, antinuclear antibody spectrum, rheumatoid factor and anticyclic citrulline polypeptide antibody were considered according to the condition Consultation in Rheumatology and Immunology department
Nervous system		Perform nervous system examination/functional evaluation as required	Individualized evaluation and follow‐up with neurology consultation as needed

^a^ It is necessary to know the symptoms, diagnosis and treatment, treatment‐related adverse reactions, past history (especially autoimmune diseases, immunodeficiency diseases, tuberculosis, viral hepatitis, organ transplantation, etc.), allergy history and family history (especially autoimmune diseases or immunodeficiency‐related family history). Ask whether there are symptoms and signs related to immune system diseases, including alopecia, photosensitivity, butterfly erythema, discoid erythema, rampant dental caries, recurrent oral ulcer and/or vulvar ulcer, uveitis, dry eyes, dry mouth, joint pain, joint swelling, inflammatory low back pain, myalgia, myasthenia, mucopurulent bloody stool, etc.

^b^ Imaging examination: chest and abdomen enhanced CT, head enhanced MRI and whole body bone imaging to evaluate the primary focus and whether there is distant metastasis; It is recommended to perform whole body PET/CT staging.

^c^ Routine blood test, blood biochemistry (AST, ALT, ALP, GGT, TBil, DBil, TP, Alb, prealbumin, LDH, Cr, BUN, Glu, k, Na, Cl, Ca, p, CO_2_ binding force, UA, AMY, LIP), coagulation (PT, APTT, Fib, lip) Inflammatory indicators: C‐reactive protein, ESR, interleukin−6, interleukin−8, interleukin−10, tumor necrosis factor‐α and ferritin.

^d^ Autoantibodies: ANA, anti‐ds‐DNA, anti‐RNP, anti‐SSA, anti‐SSB, anti‐Scl‐70, anti‐Jo‐1, antimitochondrial antibody M2 subtype (AMA‐M2); Antineutrophil cytoplasmic antibody; Anticyclic citrulline polypeptide antibody, antinuclear factor, antikeratin antibody and rheumatoid factor.

^e^ Endocrine related indicators: TSH, FT3, FT4, A‐Tg, A‐TPO; ACTH (8:00 a.m.), F (8:00 a.m.); FSH, LH, T, E2, P, PRL; IGF‐1, GH. Abbreviations: ANA, antinuclear antibody; ANCA, antineutrophil cytoplasmic autoantibody; CT, computed tomography; MRCP, magnetic resonance cholangiopancreatography; MRI, magnetic resonance imaging; PET/CT, positron emission tomography/computed tomography;

#### Preoperative evaluation of irAEs after neoadjuvant therapy

(i)Obtain a medical history; perform a preoperative physical examination and treat patients with hypertension, diabetes, and coronary heart disease. After the condition has stabilized, surgery can be considered.

(ii)Perform routine preoperative blood analyses, including biochemistry and coagulation; correct anemia, electrolyte disorders, malnutrition, coagulation disorders, and so on. Consider surgery after the condition has improved.

(iii)Perform chest imaging and electrocardiography.

(iv)Perform fiberoptic or endobronchial ultrasound bronchoscopy.

(v)Perform standard pulmonary function tests to evaluate respiratory function.

(vi)For all baseline tests, perform re‐examination as necessary for abnormal inspections.

(vii)Senior thoracic surgery experts should evaluate the indications for surgery, and if necessary, multidisciplinary consultation should be led by thoracic surgeons.

#### Postoperative evaluation

(i)Evaluate the patient's consciousness, breathing, and circulation status.

(ii)Evaluate wound healing.

(iii)Evaluate the general condition of the drainage tube.

(iv)Evaluate the patient for possible surgical complications such as difficulty in expectoration, subcutaneous emphysema, pulmonary rales, asymmetric respiratory sounds, and arrhythmia.

(v)Perform routine postoperative blood analyses, including biochemistry and coagulation, and add appropriate inspection items according to the postoperative status of the patient.

#### Routine evaluation

(i)Ask the patient about new symptoms or exacerbation of original symptoms; conduct a detailed and meticulous physical examination, including height, weight, physical strength scores (e.g., Eastern Cooperative Oncology Group Performance Status, Karnofsky Performance Status), and pain scores if necessary.

(ii)Perform a general and targeted inspection of suspected AEs before each systemic treatment cycle (see Table 4).

(iii)Imaging should generally be performed every three months after surgery to re‐evaluate the primary tumor.

### Exploration of perioperative treatment plans

A new adjuvant regimen for ICIs combined with chemotherapy has been designed following several phase III clinical studies of neoadjuvant immunotherapy in NSCLC, including the CheckMate‐816 (NCT02998528), KEYNOTE‐671 (NCT03425643), IMpower‐030 (NCT03456063), and AEGEAN (NCT03800134) trials. The same regimen also had the highest MPR/pCR rate in earlier phase I/II clinical trials. Based on these studies, neoadjuvant ICIs combined with chemotherapy should be recommended as a priority for operable patients in good physical condition (Performance Status score 0 or 1). ICI monotherapy is an important option among the many neoadjuvant therapies. For operable NSCLC with high tumor expression of PD‐L1, ICI monotherapy can be considered as neoadjuvant therapy, but the actual curative effect remains to be determined in large‐scale phase III clinical trials. Similarly, the benefits and safety of double ICI therapy with a PD‐1/PD‐L1 inhibitor and CTLA‐4 inhibitor as perioperative therapy will need to be confirmed in a large‐scale study. At present, there is no consensus “best course” of neoadjuvant immunotherapy for NSCLC. Most current clinical studies empirically employ neoadjuvant therapy for 2–4 cycles before surgery.

The phase III trials of perioperative immunotherapy for NSCLC tend to favor 3–4 cycles of chemotherapy plus immunotherapy preoperatively, and adjuvant therapy with or without chemotherapy postoperatively. Phase III clinical studies of immunotherapy for patients with stage IB–IIIA NSCLC with no preoperative treatment have seldom employed adjuvant chemotherapy plus immunotherapy administered concurrently, but have instead opted for sequential treatment. For example, the IMpower010 (NCT02386718) and ANVIL (NCT02595944) studies of stage IB‐IIIA NSCLC patients (*n* = 1280 and 903, respectively) employed sequential single‐immunotherapy maintenance after four cycles of standard postoperative adjuvant chemotherapy. However, because perioperative chemotherapy has minimal benefit and leads to only a 5% improvement in the five‐year postoperative survival rate, the ANVIL (NCT02595944), PEARLS (NCT02504372), and BR.31(NCT02273375) studies of IB–IIIA NSCLC patients all included postoperative adjuvant chemotherapy as an option, with maintenance treatment with a single immunotherapeutic drug for one year.

In summary, there is currently no high‐level medical evidence to support a specific regimen for perioperative immunotherapy. Preliminary results suggest that combination neoadjuvant immunotherapy plus chemotherapy results in a good pathological remission rate, but whether the high MPR/pCR rate can be transformed into survival will require confirmation in phase III clinical trials. For postoperative adjuvant therapy, the survival index is the most important evaluation standard, and most current postoperative adjuvant research will not be completed until after 2024. Before a consensus is reached, it will be necessary to further explore chemotherapy combined with ICIs, sequential chemotherapy and ICIs, and ICI monotherapy or dual therapy.

### Perioperative irAEs

#### Preoperative irAEs

Most neoadjuvant immunotherapy is administered in 2–4 cycles. A few phase II clinical studies with small sample sizes have examined the influence of immunotherapy on surgical outcomes. The LCMC3 study^23^ of 101 patients with early resectable NSCLC preliminarily reported an incidence of 29% grade 3–4 AEs after two cycles of preoperative atezolizumab. The most common AEs were fatigue, fever, anorexia, transaminase elevation, nausea, joint pain, flu‐like symptoms, diarrhea, pneumonia, and anemia, but overall, the treatment was well tolerated and there were no delays in surgery. The study found no significant difference in the incidence of AEs between the two groups, including grade 3–5 AEs of hypermagnesemia (4%), hypoxemia (4%), severe diarrhea (4%), and hyponatremia (4%).

#### Intraoperative conversion to thoracotomy rate

In a study to evaluate the safety of nivolumab neoadjuvant immunotherapy in patients with resectable NSCLC (stage IA–IIIA),^24^ seven of the 13 patients (53.8%) were converted to thoracotomy due to hilar inflammation or fibrosis after neoadjuvant immunotherapy. The seven patients included four with stage IA NSCLC, of whom one was converted (25%). The study reported no significant differences in operation time (228 min) and blood loss (100 ml) for patients receiving neoadjuvant chemotherapy and neoadjuvant immunotherapy.

#### Postoperative irAEs and complications

In the study^24^ of nivolumab neoadjuvant immunotherapy in 20 patients with stage IA–IIIA NSCLC, the postoperative incidence of AEs was ~30% (6/20) for atrial arrhythmias, and 5% (1/20) each for myocardial infarction, pulmonary embolism, and empyema. In the NEOSTAR study described above,^15^ patients treated preoperatively with two cycles of nivolumab exhibited postoperative complications of persistent lung leakage (22%), bronchopleural fistula (9%), empyema (4%), pulmonary infection (4%), and nonspecific pneumonia (4%). In the most recent report from the NADIM study of 41 NSCLC patients treated with nivolumab plus carboplatin and paclitaxel,^6^ the postoperative complication rate was 17.1% and included arrhythmia, persistent lung leakage, respiratory tract infection, postoperative pain, recurrent laryngeal nerve paralysis, thrombocytopenia, postoperative pulmonary infection, lower limb cellulitis, and atrial fibrillation.

These studies suggest that, overall, neoadjuvant immunotherapy for patients with operable NSCLC is relatively safe, with incidences of any‐grade and ≥3 grade irAEs of 23%–57% and 4.5%–13%, respectively. However, these are mostly phase I/II exploratory studies with small sample size, short follow‐up time, and incomplete data. Therefore, we do not yet have a complete picture of potential AEs related to neoadjuvant immunotherapy, and the results of large‐scale, prospective, and long‐term follow‐up studies are still needed. Past experience and published data have revealed that patients with advanced cancer may experience many types of irAEs that can affect their prognosis. For patients with operable lung cancer, perioperative irAEs will inevitably have a profound impact on their follow‐up treatment. Therefore, a comprehensive and standardized perioperative AE management plan can not only ensure smooth implementation of the overall treatment plan but also play a positive role in improving the clinical outcome of patients. The majority of lung cancer clinicians should consider adopting such management plans.

## TREATMENT OF IRAES

### Classification of irAEs

While activation of T cell function by ICIs might be expected to increase inflammatory AEs, the precise pathophysiological mechanisms of action of ICIs are not yet fully understood. As noted in the introduction, irAEs may result from the activity of autoreactive T cells, autoantibodies, or cytokines, among other possibilities. irAEs of various grades affecting many systems and organs throughout the body have been reported. In the majority of AE grading systems, grades 1–2 AEs are mild to moderate and do not require hospitalization; grade 3 AEs have obvious symptoms or worsening symptoms, are considered severe, and require hospitalization; grade 4 AEs have life‐threatening and/or disabling symptoms and require intensive care (Table 5).

**TABLE 5:** Immunotherapy‐related adverse event (irAE) classification

Affiliated organ or system	Disease name	Grade
Injection reaction		G1: Mild temporary reaction, no need to suspend injection, no need for special treatment G2: Suspension of infusion, immediate systemic treatment (antihistamines, NSAIDs, opioids, intravenous rehydration), drug treatment ≤24 h G3: Symptoms prolonged or recurred after initial treatment (symptoms improved significantly after treatment or symptoms still recurred after suspension of infusion); hospitalization; other medical interventions are needed G4: Life‐threatening requiring emergency intervention G5: Death
Cytokine release syndrome (CRS)		G1:T ≥ 38°C, no hypotension and hypoxemia G2:T ≥ 38°C, hypotension (without vasoactive drugs), and/or hypoxemia (nasal catheter ≤6 L/min) G3:T ≥ 38°C, hypotension, need of a vasoactive drug, and/or hypoxemia (high flow), mask, Venturi mask G4: T ≥ 38°C, hypotension, need of multiple vasoactive drugs, and/or hypoxemia (invasive or noninvasive mechanical ventilation)
Skin	Spot papule	G1: Skin lesions <10% of body surface area, asymptomatic G2: Skin lesions are between 10% and 30% BSA, with or without (itching/burning/tightness); G3–4: Skin lesions >30%, with or without (itching/burning/tightness)
	Itch	G1: Itching is limited and mild G2: Itching is intense, extensive and intermittent, and changes with scratching rash (such as edema, papule, skin shedding, exudation/crusting) G3: Intense, extensive and persistent itching; affect ADL and sleep
	Bullous pemphigoid	G1 is asymptomatic, with blisters covering 10% BSA G2 blister 10%–30% BSA, painful blister, limited ADL G3 blister >30% BSA, causing water and electrolyte disturbance, indicating ICU or burn ward
	Mossy dermatitis	G1: Skin lesion <10% of body surface area, asymptomatic G2: Skin lesions are between 10% and 30% BSA, with or without (itching/burning/tightness); G3–4: Skin lesions >30%, with or without (itching/burning/tightness)
	Psoriasis	G1: Skin lesion <10% of body surface area, asymptomatic G2: Skin lesions are between 10% and 30% BSA, with or without (itching/burning/tightness); G3–4: Skin lesions >30%, with or without (itching/burning/tightness) symptoms
	Vitiligo	G1: Skin lesion <10% of body surface area, asymptomatic G2: Skin lesions are between 10% and 30% BSA, with or without (itching/burning/tightness); G3–4: Skin lesions >30%, with or without (itching/burning/tightness) symptoms
	Capillary hyperplasia of skin	G1: Single maximum diameter ≤ 10 mm, with or without bleeding from rupture G2: Single maximum diameter > 10 mm, with or without rupture and bleeding G3: It is generalized and may be complicated with skin infection, which may require hospitalization G4: Multiple and widespread, threatening life G5: Died
	Stevens‐Johnson syndrome (SJS)/ toxic epidermal necrolysis (TEN)	G4
	Drug eruption with eosinophilia and systemic symptoms (DRESS)	G4
	Acute febrile neutropenia (Sweet)	G4
Respiratory system	Pneumonia	G1: Asymptomatic; the lesion is limited to one lobe of lung or the lung parenchyma with the lesion range less than 25% G2: New respiratory symptoms or aggravation of the original symptoms, including shortness of breath/cough/chest pain/fever, and increased oxygen inhalation conditions; G3: Severe symptoms, all lung lobes or >50% lung parenchyma involved, and limited daily activities G4: Life‐threatening respiratory damage
Digestive system	Diarrhea	G1: Less than four times G2: 4–6 times
	Colonitis	G1: No more than four times/day, no signs of systemic poisoning, normal ESR G2: >4 times/day, mild anemia, no severe abdominal pain, low fever G3–4: ≥6 times/day, with severe colic, systemic poisoning symptoms (T ≥ 37.5°C, HR ≥90 BPM, HGB < 10.5 g), ESR ≥30 mm/h, and rapid weight loss
	Elevated transaminase	G1: <3 ULN G2: 3–5 ULN G3: >5–20 ULN G4: >20 ULN
	Hyperbilirubinemia	Transaminase >3 ULN Bilirubin >1.5 ULN
	Hyperamylasemia; hyperamylasemia	G1: AMY ≤ 3 ULN + LIP ≤3 ULN G2: AMY/LIP 3–5 ULN G3–4: AMY/LIP > 5 ULN
	Acute pancreatitis	G1: Symptoms or enzymology or imaging are consistent with pancreatitis G2: Symptoms/enzymology/imaging, both of which are consistent with pancreatitis G3–4: Symptoms, enzymology, imaging or vomiting or unstable hemoglobin
Circulatory system	Pericarditis	G1: Asymptomatic, abnormal cardiac markers or ECG G2: Mild symptoms, cardiac markers and abnormal ECG G3: Color Doppler echocardiography indicated that left ventricular ejection fraction (LVEF) was less than 50% or local wall motion was abnormal. Cardiac MRI diagnosis or suspicion of myocarditis G4: Life‐threatening cardiac abnormalities such as malignant arrhythmia and cardiogenic shock
	Myocarditis	
	Myocardiopathy	
	Arrhythmia	
	Myocardial ischemia	
Endocrine system	Subclinical hypothyroidism	Thyroid stimulating hormone (TSH) increased (4–10 mIU/l), and free T4 was normal Thyroid stimulating hormone (TSH) increased (> 10 mIU/l), and free T4 was normal Thyroid stimulating hormone (TSH) is normal or decreased, free T4 is decreased, and central hypothyroidism is considered
	Hypothyroidism	G1: TSH < 10 mIU/l, asymptomatic; only clinical or diagnostic observation is needed; there is no need for treatment G2: Persistent TSH > 10 mIU/l with symptoms; affects the use of instrumental activities of daily living G3: Severe symptoms; personal self‐care ability is limited; need hospitalization G4: Life‐threatening; emergency intervention is required
	Thyrotoxemia	Thyroid stimulating hormone (TSH) decreased and free T4 was normal or increased
	Central hypothyroidism	Low TSH or inhibition with inappropriate low free T4
	Hypophysitis	G1: asymptomatic or mild symptoms G2: Moderate symptoms, able to carry out activities of daily living G3–4: severe symptoms that are medically significant or life‐threatening; self‐rational activities of daily life are limited
	Isolated adrenal hypofunction	G1: Asymptomatic or mild symptoms G2: Moderate symptoms, able to carry out activities of daily living G3–4: Severe symptoms that are medically significant or life‐threatening; self‐rational activities of daily life are limited
Nervous system	Myasthenia gravis	G1: Asymptomatic (G2 for any intracranial neuropathy) G2: Limit ADL score G3–4: Limit self‐care ability, limit walking, and limit walking or breathing with any degree of dysphagia, facial weakness, breathing machine weakness or rapid progressive symptoms
	Guillain‐Barré Syndrome	
	Peripheral neuropathy	
	Inflammatory myopathy	
	Aseptic meningitis	
	Brain fever	
	Multiple sclerosis	
	Optic neuritis	
	Transverse myelitis	
	Facioplegia	
Rheumatoid immune system	Inflammatory arthritis	G1: Mild pain with inflammation, erythema or joint swelling G2: Moderate pain with signs of inflammation, erythema or joint swelling, affecting instrumental activities of daily living G3–4: Severe pain with signs of inflammation, erythema or joint swelling; irreversible joint injury; disability; personal self‐care ability is limited
	Myositis	G1: Mild symptoms with or without pain G2: Moderate symptoms with or without pain, affecting instrumental activities of daily living G3–4: Severe symptoms with or without pain, limited self‐care ability
	Polymyalgia rheumatica	Mild: Mild pain and/or stiffness, and unlimited activities of daily living Moderate/severe: pain and/or stiffness affecting instrumental activities of daily living or self‐care ability
	Giant cell arteritis	Visual changes, headache, scalp tenderness, mandibular lameness
Blood system	Autoimmune hemolytic anemia	G1: Hgb < LLN –100 g/l G2: Hgb < 100–80 g/l G3: Hgb < 80 g/l G4: Life‐threatening, requiring emergency intervention and treatment
	Immune thrombocytopenia	G1: Platelets <100/μl G2: Platelets <75/μl G3: Platelets <50/μl G4: Platelets <25/μl
	Aplastic anemia (AA)	Moderate AA: (1) The proliferation degree of bone marrow cells is less than 30%; (2) no severe pancytopenia; (3) at least two of the three blood components are lower than normal Severe AA (SAA): (1) The proliferation degree of bone marrow cells is less than 25% or (2) bone marrow biopsy shows that the proliferation degree of cells is less than 50%, among which hematopoietic cells are less than 30%, and there are (1) reticulocyte count <40 000/μl; neutrophils <500/μl; PLT < 20 000/μl Extremely severe AA: Meet SAA standard and ANC is less than 200/μl
Kidney	Nephritis	G1: Creatinine >0.3 mg/dl; creatinine increased to 1.5–2 times the baseline level G2: Creatinine level increased to 2–3 times the baseline level G3: Creatinine level > 4.0 mg/dl; creatinine increased to >3 times baseline level G4: Life threatening; alternative treatment is needed
Eye	Uveitis	G1: Mild symptoms G2: Anterior uveitis G3: Posterior uveitis or total uveitis G4: Vision 20/200 (legally blind)
	Vogt‐Koyanagi‐Harada syndrome‐like changes	G1: Mild symptoms G2: Vision 20/40 or better G3: Vision is less than 20/40 G4: Vision 20/200 (legally blind)

### General principles of irAE treatment

(i)Adhere to the important principle of “prevention, assessment, inspection, treatment, and monitoring” for the management of ICIs to ensure early and accurate detection, diagnosis, and treatment of irAEs.

(ii)Encourage close consultation with specialists in specific diseases; complex cases or multisystem irAEs may need to be referred to tertiary medical institutions for diagnosis and treatment, and delays in the best treatment opportunity must be avoided for critically ill patients.

(iii)ICI treatment should be suspended if irAEs of grade ≥ 2 occur; treatment can then be resumed if symptoms or/and laboratory tests are reduced to grade 1 or below. For symptoms persisting for >1 week, glucocorticoid (GC) treatment should be started.

(iv)Patients with grade 3–4 irAEs should be treated with GCs, which will generally reduce most AEs to grade 1 or below over 4–6 weeks.

(v)ICI treatment should be permanently discontinued for patients with grade 4 irAEs (or endocrine irAEs that can be controlled by nonalternative therapy). Permanent discontinuation of ICIs may be considered for patients with grade ≥2 irAEs lasting for more than six weeks, or if GC therapy cannot be reduced to <10 mg prednisone (or equivalent) within 12 weeks.

(vi)For ICIs that fail to respond to at least 72 h of intravenous GC treatment, other immunomodulators or treatment regimens should be considered.

(vii)Inactivated or attenuated vaccine preparations can be administered while patients are on ICI therapy, but administration of live vaccines is not advised.

### Principles of irAE prevention

(i)For patients receiving high‐dose GC therapy (1–2 mg/kg/day), especially shock doses, or patients with high risk factors for gastrointestinal bleeding, consider adding proton pump inhibitors or H2 receptor blockers.

(ii)For patients receiving prednisone at ≥20 mg/day for ≥4 weeks, prevention of *Pneumocystis carinii* pneumonia and other fungal infections must be considered.

(iii)Long‐term GC therapy increases the risk of osteoporosis. Supplementation with oral vitamin D and calcium and monitoring of bone metabolism indexes is recommended, and antiosteoporosis drugs should be considered when necessary.

(iv)Patients on GC therapy should be educated to avoid crowded places or contact with infection sources by wearing personal protection (masks), washing hands frequently, and paying attention to food hygiene; and to avoid substantial weight gain by controlling food intake. Blood sugar, blood pressure, and electrolyte levels should be monitored.

### Principles of glucocorticoid use

GCs are steroid hormones secreted by the adrenal cortex and play important roles in regulating the development, growth, metabolism, and immune function. GCs have a spectrum of anti‐inflammatory, antiallergic, antishock, and immunoregulatory properties that are mediated through transcriptional and nontranscriptional pathways. Lipid‐soluble GCs enter the cell directly through the plasma membrane or via transporters and bind to the cytosolic GC receptor α (cGCR). GC binding induces a conformational change and dissociation of the cGCR from molecular chaperones such as heat shock protein 90 (HSP90), HSP70, HSP56, and HSP40. In the classical direct transactivation mechanism, the activated GCR translocates to the nucleus and binds to specific DNA sequences in the target genes known as positive or negative GC response elements. These interactions modulate the transcription of target genes, leading to upregulation or downregulation of various inflammatory and immunoregulatory mediators. Because alteration of protein expression at the level of gene transcription and translation take time, the classical genomic effect of GCs takes several hours or days to produce significant clinical effects. In the second nontranscriptional or transrepression mechanism, various activities of GCs and the GCR in the cytoplasm can result in physiological or pharmacological responses within seconds or minutes. Therefore, the efficacious properties and toxic side effects of different GCs may occur with distinct kinetics (Table 6). According to the pharmacokinetic characteristics of GCs, they can be divided into short‐acting, medium‐acting, and long‐acting molecules (Table 6). Most oral GCs are absorbed within 30 min and have high oral bioavailability.

**TABLE 6:** Hormone species and their pharmacokinetic characteristics

Hormone kind dosage grade	Short‐acting hormone (action time is 8–12 h)		Moderate hormone (action time 18–36 h)			Long‐acting hormone (action time is 36–54 h)		Genome effect	Nongenomic effect
	Cortisone	Hydrocortisone	Prednisone	Prednisolone	Methylprednisone	Dexamethasone	Betamethasone		
Equivalent dose	25 mg	20 mg	5 mg	5 mg	4 mg	0.75 mg	0.6 mg		
Low dose	≤37.5 mg	≤30 mg	≤7.5 mg	≤7.5 mg	≤6 mg	≤1.125 mg	≤0.9 mg	+	
Moderate dose	37.5 mg–150 mg	30 mg–120 mg	7.5 mg–30 mg()	7.5 mg–30 mg	6 mg–24 mg	1.125 mg–6 mg	0.9 mg–3.6 mg	++	+
megadose	150 mg–500 mg	120 mg–400 mg	30 mg–100 mg	30 mg–100 mg	24 mg–80 mg	6 mg–15 mg	3.6 mg–12 mg	++	+
Oversize dose	>500 mg	>400 mg	>100 mg	>100 mg	>80 mg	>15 mg	>12 mg	+++	++
Impact dose			≥250 mg	≥250 mg	>200 mg	>37.5 mg		+++	+++

The most effective treatment of irAEs depends on the early initiation of GC therapy. Overall, guidelines on GC dosage and regimen are similar in Japan and the rest of the world, although some details differ (Table 7). In general, GCs or immunomodulators are not recommended for grade 1 irAEs, and ICI therapy can be continued. ICIs should be suspended for grade ≥2 irAEs, and local or systemic treatment with a moderate dose of GC (0.5–1 mg/kg/day) is recommended. For irAEs of grade 3 or 4, ICIs should be permanently withdrawn, and systemic treatment with a large dose, or even shock dose, of a moderate‐effect GC is suggested. Immunomodulators or other treatments can be added according to persisting symptoms within 3–5 days of GC administration.

**TABLE 7:** Suggestions on initial dose of glucocorticoid for immunotherapy‐related adverse event (irAE) treatment in NCCN guidelines, ESMO guidelines, SITC guidelines and CSCO guidelines

irAE	Grade	NCCN (2020.V1)	ESMO (2017)	SITC (2017)	ASCO (2018)	CSCO (2019)
Injection reaction	Level 2–4	Application not recommended	/	Applications can be considered	/	Application not recommended
Primary irritation	Level 2	Pred 0.5–1 mg/kg/day	/	Pred 0.5–1 mg/kg/day	Pred 1 mg/kg/day (or equivalent dose)	Pred 0.5–1 mg/kg/day
	Grade 3–4	Rash: Pred 0.5–1 mg/kg/day Bullous dermatitis: Pred 1–2 mg/kg/day SJS/TEN: Pred 1–2 mg/kg/day	Grade 3: Pred 0.5–1 mg/kg/day Grade 4: MP 1–2 mg/kg/day	Pred 1–2 mg/kg/day	Pred 1–2 mg/kg/day (or equivalent dose)	Pred 0.5–1 mg/kg/day
Lung toxicity	Level 2	Pred 1–2 mg/kg/day	Pred 1 mg/kg/day	MP 1 mg/kg/day	Pred 1–2 mg/kg/day	MP 1–2 mg/kg/day
	Grade 3–4	MP 1–2 mg/kg/day	MP 2–4 mg/kg/day	MP 2 mg/kg/day	MP 1 g/day, 5 days	MP 2 mg/kg/day
Gastrointestinal toxicity (diarrhea/colitis)	Level 2	Pred 1–2 mg/kg/day	Pred 0.5–1 mg/kg/day	Pred 1 mg/kg/day	Pred 1 mg/kg/day	Pred 1 mg/kg/day
	Grade 3–4	MP 1–2 mg/kg/day	MP 1–2 mg/kg/day	MP 1–2 mg/kg/day	Pred 1–2 mg/kg/day	MP 2 mg/kg/day
Hepatotoxicity	Level 2	Pred 0.5–1 mg/kg/day	Pred 1 mg/kg/day	Pred 0.5–1 mg/kg/day	Pred 0.5–1 mg/kg/day	Pred 0.5–1 mg/kg/day
	Grade 3–4	Grade 3: Pred 1–2 mg/kg/day Grade 4: Pred 2 mg/kg/day	Grade 3: Bilirubin /INR/albumin is normal MP 1 mg/kg/day; Elevated bilirubin/increased /INR/decreased albumin MP 2 mg/kg/day Grade 4: MP 2 mg/kg/day	Pred 1–2 mg/kg/day	Grade 3: Pred 1–2 mg/kg/day Grade 4: Pred 2 mg/kg/d	MP 1–2 mg/kg/day
Cardiac toxicity	Level 2	/	/	Case‐by‐case is recommended for suspected myocarditis	Pred 1–2 mg/kg/day	/
	Grade 3–4	MP 1 g/day, 3–5 days	/	MP 1 mg/kg/day	MP 1 g/day	MP 1 g/day, 3–5 days
Hypophysitis	Level 1	Pred 1–2 mg/kg/day	/	/	HCT 15–30 mg/day	Pred 1–2 mg/kg/day
	2 degrees		Pred 0.5–1 mg/kg/day	HCT 10 mg/m^2^	20 mg/day Pred or 30–50 mg/day HCT	
	Grade 3–4		MP 1 mg/kg/day	Pred 1 mg/kg/day	Pred 1–2 mg/kg/day	
Arthritis	Level 2	Pred 0.5 mg/kg/day	Pred 10–20 mg		Pred ≤10 mg/day	Pred 0.5 mg/kg/day
	Grade 3–4	Pred 1 mg/kg/day	MP 0.5–1 mg/kg/day		Pred 0.5–1 mg/kg/day	Pred 1 mg/kg/day
Neurotoxicity	Level 2	MG: Pred 20 mg/day GBS: MP 1 g/day, 5 days Peripheral neuropathy: Pred 0.5–1 mg/kg/day Aseptic meningitis: Pred 0.5–1 mg/kg/day Encephalitis: MP 1–2 mg/day	GBS: MP 1–2 mg/kg/day Peripheral neuropathy: Pred 0.5–1 mg/kg/day Aseptic meningitis: Pred 0.5–1 mg/kg/day	MP 0.5–1.0 mg/kg/d	MG: Pred 1–1.5 mg/kg/day; GBS: MP 2–4 mg/kg/day; Peripheral neuropathy: Pred 0.5–1 mg/kg/day Autonomous neuropathy: Pred 0.5–1 mg/kg/day Aseptic meningitis: Pred 0.5–1 mg/kg/day Encephalitis: MP 1–2 mg/day	MG: Pred 1–1.5 mg/kg/day; GBS: MP 2–4 mg/kg/day; Aseptic meningitis: 1 mg/kg/day Encephalitis: MP 1–2 mg/kg/day
	Grade 3–4	MG: MP 1–2 mg/kg/day GBS: MP 1 g/day, 5 days Peripheral neuropathy: MP 1 g/day, 5 days Aseptic meningitis: Pred 1–2 mg/kg/day Encephalitis: MP 1 g/day, 3–5 days Transverse myelitis: MP 1 g/day, 3–5 days	Peripheral neuropathy: Pred 2 mg/kg/day Aseptic meningitis: MP 1–2 mg/kg/day Transverse myelitis: MP 2 mg/kg/day (1 g/day can be considered)	MP 1–2 mg/kg/day	MG:MP 1–2 mg/kg/day; GBS:MP 2‐4 mg/kg/day; Peripheral neuropathy: MP 2–4 mg/kg/day Autonomic neuropathy: MP 1 g/day, 3 days Aseptic meningitis: MP 1 g/day Encephalitis: MP 1 g/day, 3–5 days; Transverse myelitis: MP 1 g/day, 3–5 days	MG: MP 1–2 mg/kg/day; GBS: MP 2–4 mg/kg/day; Aseptic meningitis: MP 1 mg/kg/day Encephalitis: MP 1–2 mg/kg/day; Transverse myelitis: 1 g/day, 3–5 days
Anemia	Level 2	–	–	–	Pred 0.5–1 mg/kg/day	AIHA: Pred 0.5–1 mg/kg/day AA: Hormones are not recommended
	Grade 3–4	–	–	–	Pred 1–2 mg/kg/day	AIHA: 1–2 mg/kg/day AA: Not recommended
Thrombocytopenia	Level 2	–	–	–	Pred 1 mg/kg/day	Pred 0.5–2 mg/kg/day
	Grade 3–4	–	–	–	Pred 1–2 mg/kg/day	Pred 1–2 mg/kg/day
Renal toxicity	Level 2	Pred 0.5–1 mg/kg/day	Pred 0.5–1 mg/kg/day	Start to use, and the dose will be formulated individually	Pred 0.5 mg/kg/day	Pred 0.5–1 mg/kg/day
	Grade 3–4	Pred 1–2 mg/kg/day	MP 1–2 mg/kg/day		Pred 1–2 mg/kg/day	MP 1–2 mg/kg/day
Ocular toxicity	Grade 3–4	Systemic glucocorticoid (ophthalmic specialist opinion)		General and local glucocorticoids (ophthalmic specialist opinion)	MP 1–2 mg/kg/day	General and local glucocorticoids (ophthalmic specialist opinion)

Abbreviations: AA, aplastic anemia; AIHA, autoimmune hemolytic anemia; GBS, Guillain‐Barre syndrome; HCT, hydrocortisone; MG, myasthenia gravis; MP, methylprednisolone; pred, prednisone.

### Intravenous injection of immunoglobulin (IVIG)

IVIG consists of blood components, mainly immunoglobulins, purified from pooled normal human plasma, and it is used to treat a variety of disorders. The mechanisms of action of IVIG are complex, but their immunomodulatory activities depend predominantly on the portion of the antibody involved. The antigen‐binding portion of immunoglobulins is composed of the variable regions of the two light and two heavy chains and is known as the F(ab')2 region, whereas the opposite termini of the two heavy chains make up the constant (Fc) portion of the molecule. IVIG‐mediated immunoregulation may occur via antigen binding by the F(ab')2 domains or Fc receptor binding by the Fc domains, and can result in inhibition of pathogenic autoantibody production, complement production, T cell activation, and cytokine production, among other mechanisms. IVIG is widely used in the treatment of various severe irAEs and autoimmune/inflammatory diseases, including Guillain–Barré syndrome, myasthenia gravis, bullous rash, Stevens‐Johnson syndrome, toxic epidermal necrolysis, drug rash with eosinophilia and systemic symptoms, thrombocytopenia, and others.

For the first treatment, IVIG can be administered at 2 g/kg several times over 3–5 days. At present, a widely used course of treatment in the clinic is 400 mg/kg/day for 3–5 days, and this can be repeated if the irAE recurs. IVIG should be administered slowly, starting at 1 ml/min and not exceeding 3 ml/min, and should not be mixed with other drugs. Once the vial is opened, it should be infused at once and unused portions discarded.

IVIG is a relatively safe drug with a low (1%) incidence of side effects. Common AEs include headache, back pain, nausea, vomiting, diarrhea, facial flushing, fever, chills, shortness of breath, chest tightness, hypotension, hypertension, and rash. AEs are mostly transient, usually occur during the first or second infusion, and are generally related to rapid intravenous administration and the use of preparations from different manufacturers. A slow infusion rate can alleviate such reactions. In rare cases, a low dose of GC or an antihistamine should be given 30 min before infusion. Because IVIG contains a small amount of IgA, its use in patients with congenital IgA deficiency should be strictly monitored/prohibited due to the risk of allergic reactions.

### Other therapeutic drugs and methods

The use of long‐term medium‐dose or high‐dose GCs for perioperative therapy may increase infection or delay healing. After multidisciplinary discussion, one of several alternative therapeutic methods may be implemented for the prompt treatment of severe irAEs.

#### TNF‐α inhibitors

(i) For severe irAEs that do not respond to GC treatment within 48–72 h, treatment with TNF‐α inhibitors can be started immediately. Infliximab 5 mg/kg is a standard treatment, and there are also case reports using adalimumab and etanercept.

(ii) Patients receiving GC and infliximab or FDA‐approved biological analogues should be closely monitored and followed up. A second anti‐TNF‐α treatment can be considered as needed; if so, it can be administered twice at two weeks and six weeks after the first inhibitor.

(iii) Because infliximab could potentially reactivate HBV and HCV, patients should be screened for HBV and HCV before treatment with a TNF‐α inhibitor, and HBV/HCV carriers should be monitored during and for several months after treatment.

(iv) Similarly, infliximab may also reactivate tuberculosis, and patients should therefore be tested before initiation of TNF‐α inhibitors. If treatment is urgent, there is no need to wait for the tuberculosis test results.

#### IL‐6 receptor (IL‐6R) inhibitors

(i) For severe irAEs that do not respond to GC treatment within 48–72 h, an IL‐6R inhibitor (e.g., tocilizumab 4–8 mg/kg) can be started immediately. Treatment can be repeated 8 h later as needed, with a maximum of three doses in 24 h.

(ii) IL‐6R inhibitors should be used with caution in patients with chronic or recurrent infections. For patients with tuberculosis, invasive fungal, bacterial, viral, and other opportunistic infections, adequate anti‐infection treatment should be given before tocilizumab administration. Hematological indexes and liver and kidney function should be closely monitored during treatment.

#### Anti‐CD20 monoclonal antibody

CD20 is a transmembrane protein encoded by the *MS4A1* gene and is expressed on the surface of B lymphocytes. CD20 is a marker of pre‐B to mature B cells, but it is not expressed on hematopoietic stem cells, progenitor B cells, or mature plasma cells. The mechanism of tumor cell killing by anti‐CD20 monoclonal antibodies (e.g., rituximab) includes antibody‐ and complement‐dependent cell mediated cytotoxicity and direct intracellular signaling through CD20 that affects cell growth, cell cycle progression, and apoptosis.

At present, the NCCN guidelines recommend rituximab for the treatment of GC‐resistant bullous dermatitis at 1000 mg once every two weeks for four weeks followed by 500 mg every 12 or 18 months. For the treatment of refractory nerve damage (myasthenia gravis, aseptic meningitis, encephalitis), rituximab is recommended at 375 mg/m^2^ once weekly for four weeks or 500 mg/m^2^ once every two weeks for four weeks.

#### Mycophenolate mofetil (MMF)

MMF is a prodrug that is converted to mycophenolic acid after oral consumption. The active metabolite inhibits hypoxanthine nucleoside phosphate dehydrogenase, which reduces guanine nucleotide synthesis and selectively inhibits the proliferation and function of T and B lymphocytes, resulting in suppression of the immune response.

For the treatment of irAEs such as refractory hepatitis, pneumonia, and bullous diseases, MMF can be administered twice at 1–2 g/day orally, and the amount can be adjusted in consultation with specialists according to changes in the patient's symptoms. Gastrointestinal reactions are possible side effects of MMF and can be alleviated by drug dose reduction or discontinuation. MMF has teratogenic effects and long‐term use can lead to opportunistic infections.

#### Cyclosporine A

Cyclosporine is an 11‐amino acid cyclic polypeptide originally identified in fungal extracts. The molecule binds intracellularly to cyclophilin to form a complex that modulates mitochondrial activity and inhibits calcineurin, which is crucial for IL‐2 production. Thus, cyclosporine not only inhibits T cell function but also affects B cell differentiation.

Current guidelines for the treatment of aplastic anemia, refractory kidney damage, and refractory nerve damage caused by ICIs are two doses of 4–5 mg/kg/day taken orally 12 h apart, followed by a slow decrease to 2–3 mg/kg/day upon improvement of the irAE.

For patients with elevated serum creatinine, an initial dose of 2.5 mg/kg/day should be used and reduced to 0.5–1.0 mg/kg/day if serum creatinine rises to 30% higher than baseline during treatment. The plasma concentration of cyclosporine should be monitored carefully to ensure it remains within the safety window of 100–200 ng/ml.

Cyclosporine can cause changes in the structure and function of tubulointerstitial and renal blood vessels, leading to nephrotoxicity such as renal interstitial fibrosis, hyaline degeneration of blood vessels, and glomerulosclerosis. Acute nephrotoxicity, which is closely associated with the decline in hemodynamics, can be reversed slowly after drug reduction or withdrawal. Cyclosporine should not be administered to patients with varicella, herpes zoster, and other viral infections.

#### Plasma exchange (plasmapheresis)

During plasma exchange, plasma is removed from the patient's whole blood by membrane or centrifugal separation to remove pathogenic factors (e.g., toxic drugs, cytokines, inflammatory mediators). Normal plasma or other substitutes are then added back and the reconstituted blood is reinfused into the patient. Plasma exchange can thus temporarily restore immune function by removing factors that inhibit cellular and humoral immunity. While plasma exchange is not an etiological treatment for most diseases, it can rapidly reduce the concentration of pathogenic factors, including drugs, thus providing at least temporary relief from the disease‐ or irAE‐related damage. Current NCCN guidelines recommend plasmapheresis, usually as second‐line treatment, for nervous system diseases such as myasthenia gravis, Guillain–Barré syndrome, immune encephalitis, transverse myelitis.

The success rate of plasmapheresis for the treatment for severe or rapidly progressing nervous system irAEs is variable. For example, hypotension may occur within 1 h after the start of the cardiopulmonary bypass due to excessive volume or low colloid osmotic pressure of the reinfusion. Because of the requirement for heparin anticoagulation, bleeding, hematoma, and gastrointestinal bleeding may occur at the puncture site after 30 min of cardiopulmonary bypass. Plasma separation and pipeline blockage may occur during cardiopulmonary bypass in elderly patients, patients with insufficient heparin dosage or poor blood flow, and upon blood flow slowing or interruption. Patients allergic to heparin and protamine should not undergo plasma exchange.

#### Other drugs

Other drugs, including cyclophosphamide, methotrexate, sulfasalazine, leflunomide, and eltrombopag, are also used to treat irAEs. However, their use has mostly been reported as individual case studies, and there is no consensus or opinion on diagnosis and treatment. After multidisciplinary discussion, these drugs may be useful for the treatment of some refractory irAEs in clinical practice.

## MONITORING OF IRAES

Once ICI treatment is started, the possibility of irAEs should be monitored throughout the patient's care, including during dynamic symptom management and examinations during treatment, and long‐term follow‐up after treatment.

### Monitoring during and after ICI treatment

Comprehensive evaluations should be performed during ICI treatment to ensure the early detection and prompt treatment of irAEs (Table 4). Some immunotherapy‐related toxicities may not emerge until after treatment cessation, especially those affecting renal and pituitary function. Current guidelines suggest that patients should be monitored and followed up for at least 1 year after ICI treatment.

### Monitoring after treatment with GCs and other immunomodulators

As for ICIs, regular patient evaluation is necessary to detect symptoms and signs of irAEs after treatment with GCs or other immunomodulators; this is especially important due to the potential for opportunistic infections. Current guidelines suggest that symptoms and signs should be monitored for early indicators of irAEs at least every 72 h, and the treatment plan should be adjusted accordingly. For critically ill patients, the evaluation interval should be further reduced to every 24 or 48 h.

The risk of side effects in patients receiving GCs correlates positively with the GC dose and course, with the lowest risk occurring with low‐dose and short‐duration regimens. Upon first use of GCs, neuropsychiatric symptoms such as water and sodium retention, electrolyte disturbance, increased heart rate, and increased blood pressure may emerge after about three days; increased blood sugar at ~1 week; opportunistic infections at ≥3 weeks; fungal infections and osteoporosis at ≥8 weeks; and endocrine diseases such as Cushing's syndrome and adrenal cortex function suppression at >12 weeks. Activation of new or latent infection must be closely monitored during treatment with GCs and immunomodulators (Table 8).

**TABLE 8:** Clinical diagnosis and treatment suggestions of glucocorticoid (GC) and immunosuppressant in the treatment of complicated infection

Infection	Commonly used clinical examination	Commonly used clinical treatment suggestions
Bacterial contamination	Blood routine and inflammatory indicators (ESR, CRP, PCT); Pathogen smear, culture and DNA‐NGS (sputum, urine, stool, peripheral blood, catheter blood, throat swab); imaging to determine the infection focus; for lung infection, bronchoscope brush and lavage fluid smear, culture and DNA‐NGS sequencing can be considered; for other solid organ infections, biopsy tissue can be considered for pathogen detection	According to the nosocomial flora, we can choose antibiotics according to the results of drug sensitivity
*Mycobacterium tuberculosis*/non‐Mycobacterium tuberculosis	PPD test; peripheral blood: TB‐Ab, T‐Spot. TB; sputum/bronchoscopy extract: acid‐fast staining, weak acid‐fast staining, mycobacterial culture, TB/NTM nucleic acid determination, rapid molecular identification (X‐pert) of tuberculosis and rifampicin resistance; focal tissue: the pathogen sent for inspection after grinding; Chest imaging	TB: antituberculosis treatment NTM: Choose drugs according to strains
*Clostridium difficile* infection	Namely: determination of *Clostridium difficile* toxin and culture of *Clostridium difficile*; consider colonoscopy and biopsy when necessary	Metronidazole *: taken orally, 250 mg,t.i.d.–q.i.d. Vancomycin *: oral, 125 mg, q.i.d. The general course of treatment is 7–14 days
Fungal infection	Peripheral blood: routine blood test/G test/GM test/blood culture; sputum/bronchoscopic aspiration/bronchoalveolar lavage fluid: fungal smear, fungal culture, pathogenic DNA‐NGS; urine culture; chest imaging	Empirical antifungal therapy, followed by antifungal therapy according to bacteria
HBV/HCV infection	Five items of hepatitis B, HBV‐DNA; HCV‐Ab, HCV‐RNA; Liver function	Combined with the specialist opinion of infectious diseases, antiviral treatment
HIV infection	HIV‐Ab, HIV‐RNA, T cell subsets	Combined with the specialist opinion of infectious diseases, antiviral treatment
CMV infection	Peripheral blood: CMV‐DNA, cytomegalovirus antigenemia (CMVpp65); if CMV enteritis, improve colonoscopy and biopsy, CMV inclusion body can be seen on pathology	Ganciclovir*: intravenous, 5 mg/kg, q. 12 h*14 days → qd*7 days (creatinine clearance rate > 60 ml/min*kg) Sodium foscarnet*: intravenous, 60 mg/kg, q. 8 h*14 days → 90 mg/kg, qd*7 days (creatinine clearance rate is normal)
*Pneumocystis carinii* infection	Peripheral blood: routine blood test (absolute value of lymphocytes), G test, LDH; sputum/bronchoscope aspirate/bronchoscope lavage fluid: silver hexamine staining, PCP‐DNA, pathogenic DNA‐NGS; chest imaging	Compound sulfamethoxazole* (SMZ 75 mg/kg/day + tmp 15 mg/kg/days) for 2–3 weeks; it is recommended to add GC 40 mg q. 12 h* 5 days → 40 mg qd* 5 days → 20 mg qd* 11 days for severe patients (patients with sulfonamide allergy may consider desensitization treatment or use echinocandin antibiotics)

* The commonly used drugs and dosage in clinic should be adjusted according to the infection sites, liver and kidney function and specialist opinions.

## CONFLICT OF INTEREST

The authors have no potential conflicts of interest to disclose.

## Members of the Editorial Board

### Group Leader

Li Zhang (Peking Union Medical College Hospital), Nan Wu (Peking University Cancer Hospital)

### Invited Consultants and Experts

Chun‐Xue Bai (Zhongshan Hospital, Fudan University), Liang‐An Chen (The First Medical Center of Chinese PLA General Hospital), Jun Liang (Peking University International Hospital), Liu Qian (Chinese Journal of Lung Cancer), Jie Wang (State Key Laboratory of Molecular Oncology, National Cancer Center, National Clinical Research Center for Cancer, Cancer Hospital, Chinese Academy of Medical Sciences and Peking Union Medical College), Yi‐Long Wu (Guangdong Lung Cancer Institute, Guangdong Provincial People's Hospital, Guangdong Academy of Medical Sciences), Feng‐Chun Zhang (Peking Union Medical College Hospital), Shu‐Yang Zhang (Peking Union Medical College Hospital)

### Members of the Writing Experts

Yu‐Zhou Guan (Peking Union Medical College Hospital), Xiao‐Xiao Guo (Peking Union Medical College Hospital), Chun‐Xia He (Peking Union Medical College Hospital), Yue Li (Peking Union Medical College Hospital), Nai‐Xin Liang (Peking Union Medical College Hospital), Shao‐Lei Li (Peking University Cancer Hospital), Fang‐Liang Lu (Peking University Cancer Hospital), Chao Lv (Peking University Cancer Hospital), Wei Lv (Peking Union Medical College Hospital), Xiao‐Yan Si (Peking Union Medical College Hospital), Han‐Ping Wang (Peking Union Medical College Hospital), Jiang‐Shan Wang (Peking Union Medical College Hospital), Shi Yan (Peking University Cancer Hospital), Hua‐Xia Yang (Peking Union Medical College Hospital), Hui‐Juan Zhu(Peking Union Medical College Hospital), Jun‐Ling Zhuang (Peking Union Medical College Hospital) and Ming‐Lei Zhuo (Peking University Cancer Hospital)

### Penner

Jun Ni (Peking Union Medical College Hospital), Miao Huang (Peking University Cancer Hospital)

### Secretary

Xiao‐Xia Cui (Peking Union Medical College Hospital)

### Members of Consultant Expert Group

Chun Chen (Fujian Medical University Union Hospital), Jun Chen (Tianjin Medical University General Hospital, Tianjin Key Laboratory of Lung Cancer Metastasis and Tumor Microenvironment, Tianjin Lung Cancer Institute), Wen‐Tao Fang (Shanghai Chest Hospital, Shanghai Jiao Tong University), Shu‐Geng Gao (National Cancer Center, National Clinical Research Center for Cancer, Cancer Hospital, Chinese Academy of Medical Sciences and Peking Union Medical College), Jian Hu (The First Affiliated Hospital, Zhejiang University School of Medical), Tao Jiang (Tangdu Hospital, Fourth Military Medical University), Shan‐Qing Li (Peking union medical college hospital), He‐Cheng Li (Ruijin Hospital, Shanghai Jiao Tong University School of Medicine), Yong‐De Liao (Union Hospital, Tongji Medical College, Huazhong University of Science and Technology), Yang Liu (The First Medical Center of Chinese PLA General Hospital), De‐Ruo Liu (China‐Japan Friendship Hospital), Hong‐Xu Liu (Cancer Hospital of China Medical University, Liaoning Cancer Hospital & Institute), Jian‐Yang Liu (Jilin Cancer Hospital), Lun‐Xu Liu (West China Hospital, Sichuan University), Meng‐Zhao Wang (Peking Union Medical College Hospital), Chang‐Li Wang(Tianjin Medical University Cancer Institute and Hospital, National Clinical Research Center for Cancer, Key Laboratory of Cancer Prevention and Therapy, Tianjin's Clinical Research Center for Cancer), Fan Yang (Peking University People's Hospital), Yue Yang (Peking University Cancer Hospital), Lan‐Jun Zhang (Sun Yat‐Sen University Cancer Center), Xiu‐Yi Zhi (Xuanwu Hospital, Capital Medical University), Wen‐Zhao Zhong (Guangdong Lung Cancer Institute, Guangdong Provincial People's Hospital, Guangdong Academy of Medical Sciences)
